# Tumors of the broad ligament: what and when to suspect such rare location

**DOI:** 10.1590/0100-3984.2019.0073

**Published:** 2020

**Authors:** João Diogo Oliveira, Teresa Margarida Cunha, Andreia Tereso

**Affiliations:** 1 Centro Hospitalar de Lisboa Ocidental, Lisboa, Portugal; 2 Instituto Português de Oncologia de Lisboa Francisco Gentil, Lisboa, Portugal; 3 Hospital Professor Doutor Fernando Fonseca, Lisboa, Portugal

**Keywords:** Tumor, Broad ligament, Female pelvis, Magnetic resonance imaging, Tumor, Ligamento largo, Pelve feminina, Ressonância magnética

## Abstract

Although secondary involvement of the broad ligament by malignant tumors arising elsewhere in the abdomen and pelvis is common, primary tumors in this location are rare. Tumors of the broad ligament can be of mesenchymal and mixed nature, such as leiomyoma, the most common neoplasm; epithelial tumors of Müllerian type, imposing a challenge to differentiate them from other adnexal masses; unique tumors from mesonephric origin; and tumor-like lesions. Most neoplasms in this region, whether benign or malignant, usually present clinically with vague symptoms and are often discovered during a routine gynecological examination. Suspicion of such location and knowledge of the potential range of lesions of this region may allow for planning minimally invasive surgical interventions. To be considered tumor from the broad ligament, it should not be connected with either the uterus or the ovary. Thus, the imaging approach to establish the differential diagnosis includes excluding an ovarian, uterine, or tubal origin by recognizing these separately and by rebutting imaging clues pointing to these origins. This pictorial essay reviews some of the imaging findings that may suggest such location and presents some of the possible differential diagnoses by means of illustrative confirmed cases.

## Abstract (pt)

Embora o envolvimento secundário do ligamento largo por tumores malignos abdominais e pélvicos seja comum, os tumores primários são raros. Os tumores do ligamento largo podem ser de natureza mesenquimal e mista, como o leiomioma, que é a neoplasia mais comum; tumores epiteliais do tipo mülleriano, constituindo um desafio diferenciá-los de outras massas anexiais; tumores únicos de origem mesonéfrica; e lesões tumor-*like*. A maioria das neoplasias nessa região, seja benigna ou maligna, geralmente apresenta-se com sintomas clínicos vagos, sendo frequentemente apenas descobertos durante exame ginecológico de rotina. A suspeita de tal localização e o conhecimento do potencial de lesões dessa região podem permitir o planejamento de intervenções cirúrgicas minimamente invasivas. Para ser considerado tumor do ligamento largo, este não deve de forma alguma estar relacionado com o útero, o ovário ou a trompa e, portanto, a abordagem radiológica do diagnóstico diferencial inclui excluir uma origem ovariana, uterina ou tubária, reconhecendo-os separadamente e refutando os sinais radiológicos apontando para essas origens. Este ensaio iconográfico revisa alguns dos achados radiológicos que podem sugerir tal localização e apresenta alguns dos possíveis diagnósticos diferenciais, por meio de casos ilustrativos confirmados.

## INTRODUCTION

During routine clinical practice, radiologists are often confronted with a wide range of pelvic diseases, and, among tumors, rarely some arise from the broad ligaments. Thus, one should be familiar with the spectrum of pathology and radiological findings that may suggest such origin.

## ANATOMY AND EMBRYOLOGY

**Figure 1:**
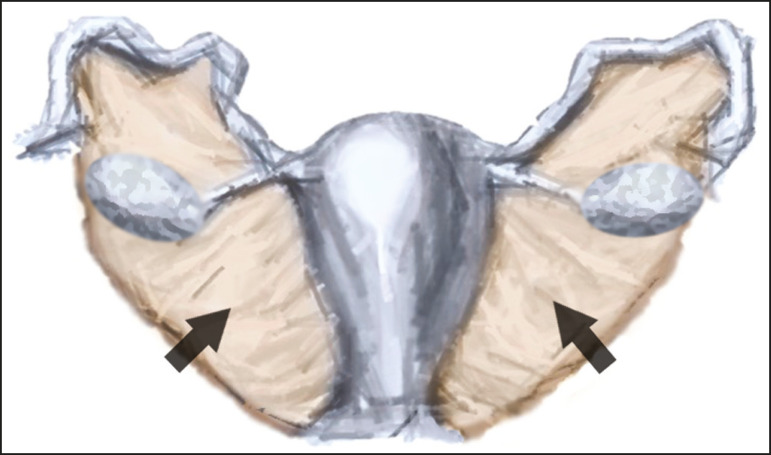
Broad ligament illustration.

The broad ligaments are the folds of the parietal peritoneum that reflect over the feminine genital tract, extending from the lateral aspect of the uterus to the pelvic wall (Figure 1). They are a double-layered sheet of mesothelial cells continuous with each other, and between them is the extraperitoneal tissue, referred to as the parametrium, which comprises the connective tissue, smooth muscles, and neurovascular elements. Embryonic remains are also normal components and may give rise to some of the unique neoplasms that can occur there, with mesonephric (Wolffian) duct remnants and paramesonephric (Müllerian) remnants being observed adjacent to the fallopian tube and near the ovary, respectively^(1)^. Other incidental tissues may also exist, such as heterotopic hilar cell clusters or adrenal cortical rests, based on neoplasms reported on the medical literature^(2)^.

## CLINICAL FEATURES AND MANAGEMENT

Although secondary involvement of the broad ligament by malignant pelvic tumors is common, primary tumors in this location are rare, and review of the medical literature reveals mostly case reports. Most neoplasms in this region, whether benign or malignant, usually present clinically with insidious and nonspecific symptoms, being often only incidentally discovered during a gynecological examination for lower abdominal discomfort or pain^(2)^. Suspicion of such location may allow for planning minimally invasive surgical interventions, with organ-preserving techniques in young patients.

Surgical removal is recommended for symptomatic relief and when there is an impingement on nearby structures, on suspected malignancy, and when pedunculated tumors are at risk of torsion.

## IMAGING FINDINGS

**Figure 2:**
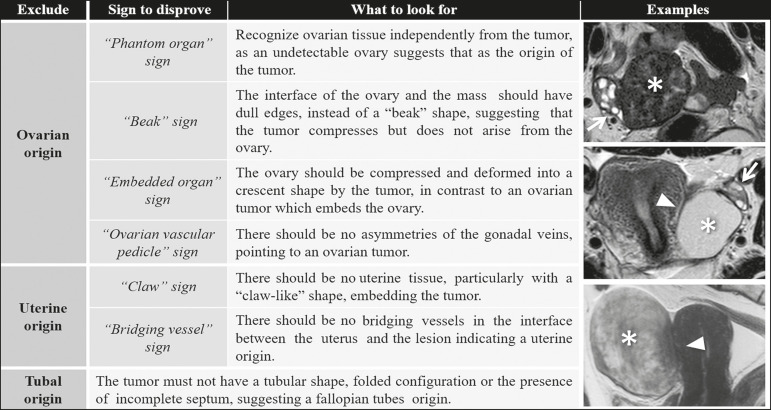
Imaging clues that may indicate a broad ligament tumor by excluding an ovarian or uterine origin. On the examples presented, the tumor is marked by asterisk, the interface with the uterus by triangle, and the ovaries by arrow.

To be considered tumor from the broad ligament, it must occur on or in the broad ligament, but be completely separated from, and in no way connected, with either the uterus or the ovary, as proposed by Gardner et al.^(3)^. Thus, the differential diagnosis approach comprises excluding an ovarian, uterine, or tubal origin by recognizing the ovary and the uterus separately and by rebutting imaging clues pointing to their origin^(4,5)^, as presented in Figure 2.

Transvaginal ultrasound, as the first-line study, can suggest broad ligament tumor when it allows clear visual separation of the tumor from the uterus and ovaries, but magnetic resonance imaging (MRI), with its multiplanar imaging capabilities, can be extremely useful for differentiating broad ligament tumors from masses of ovarian or tubal origin. Other information provided by the MRI, such as tumor size and presence of any metastatic lesion or lymph nodes, are important findings for preoperative planning and counseling, making it the gold standard imaging modality. Due to the location and size of several of these tumors, surgery is challenging, specifically since the surrounding organs such as the ureters, ovaries, intestines, and bladder may be at risk. Hence, it is important to determine the association between these tumors and organs to help guide surgery.

Commonly though, the diagnosis is difficult to be established preoperatively, being clinically and imagiologically interpreted as adnexal or uterine tumor, with the diagnosis only established during surgery or at the histopathological evaluation.

## DIFFERENTIAL DIAGNOSIS

Despite their rarity, the differential diagnoses of the tumors vary, which can be divided in epithelial tumors of Müllerian type, mesenchymal and mixed tumors, miscellaneous tumors, tumor-like lesions, and secondary tumors^(6)^. The differential diagnoses of the presented cases were histologically confirmed.

### Leiomyoma

**Figure 3:**
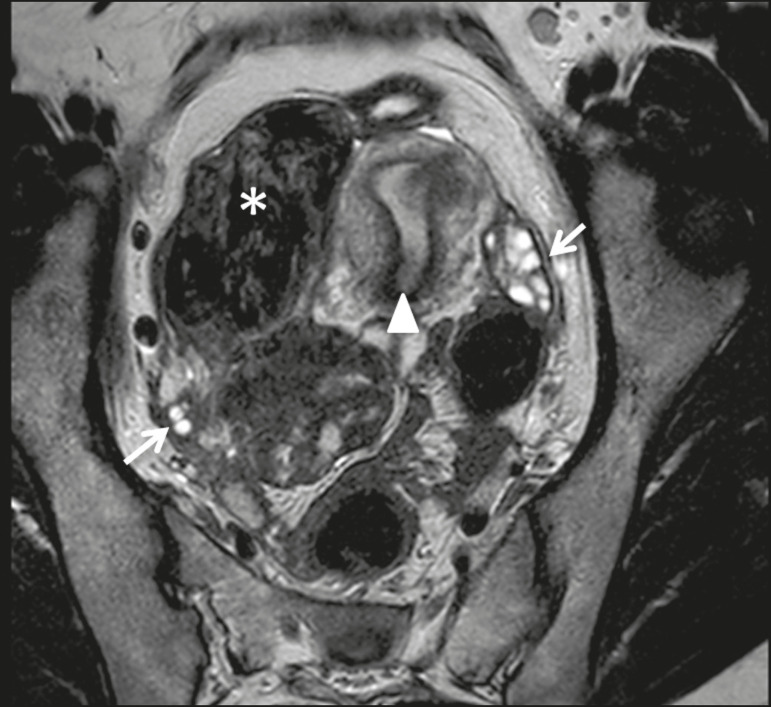
Leiomyoma of the broad ligament in a 30-year-old woman. Axial T2- weighted imaging showing a hypointense tumor (asterisk) demarked from both ovaries (arrows) and the uterus (triangle).

**Figure 4:**
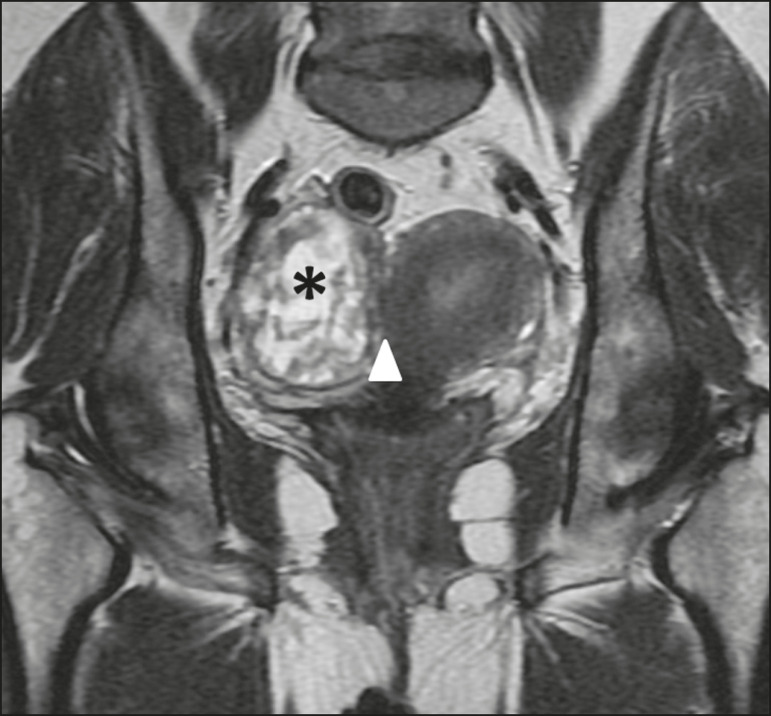
Leiomyoma of the broad ligament with cystic degeneration in a 47-yearold woman. Axial T2-weighted imaging showing a tumor with cystic areas as portions of high T2 signal (asterisk), clearly demarked from the uterus (triangle).

**Figure 5:**
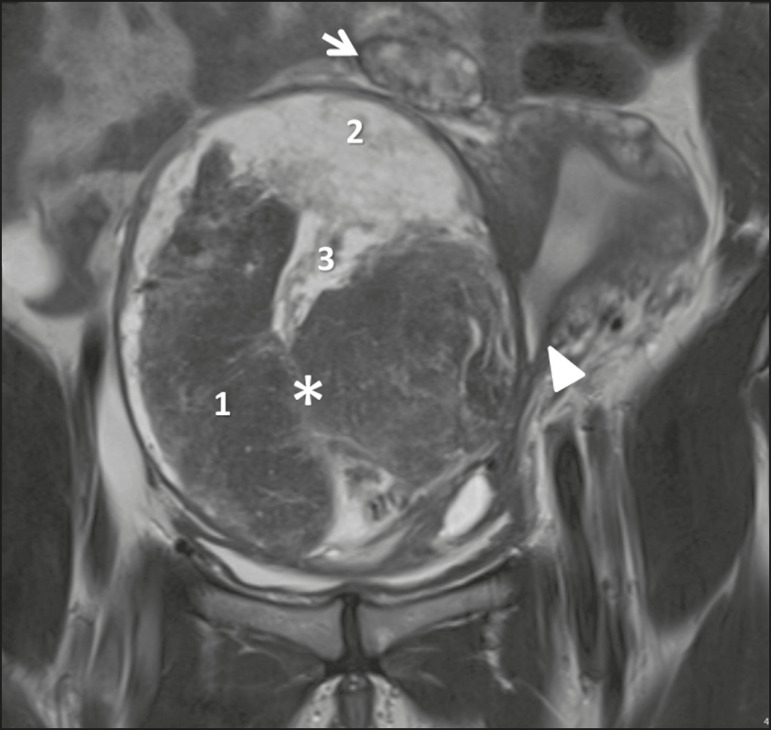
Leiomyoma of the broad ligament with hydropic degeneration in a 43-year-old woman. Coronal T2-weighted imaging showing a markedly heterogeneous tumor (asterisk) with an inferior solid component, displaying isosignal to the muscle in T2 (1) and a superior part comprising multiple large cystic areas showing high T2 signal (2), with cord-like solid components (3), which is clearly demarked from the right ovary (arrow) and the uterus (triangle).

Leiomyoma (Figures 3, 4, and 5) is the most common neoplasm observed in the broad ligament, and among them the most common extrauterine site, with a prevalence < 1%. This tumor can arise from any tissue containing smooth muscle cells and arise in the ligaments and connective tissue surrounding the ovarian and uterine vessels in the broad ligament. Tumors observed in the broad ligament are identical to those observed in the uterus and are diagnosed when it is clearly separated from the uterus. However, it may be difficult to differentiate “true” from “false” broad ligament tumor, as is tumors that arise from the lateral wall of the uterine corpus/cervix and bulge outward between the layers of the broad ligament. These benign tumors are usually asymptomatic. However, if these tumors are large, the anatomy of the pelvis is distorted and potentially compresses the ureter, leading to hydronephrosis^(2,4)^.

### Leiomyosarcoma

**Figure 6:**
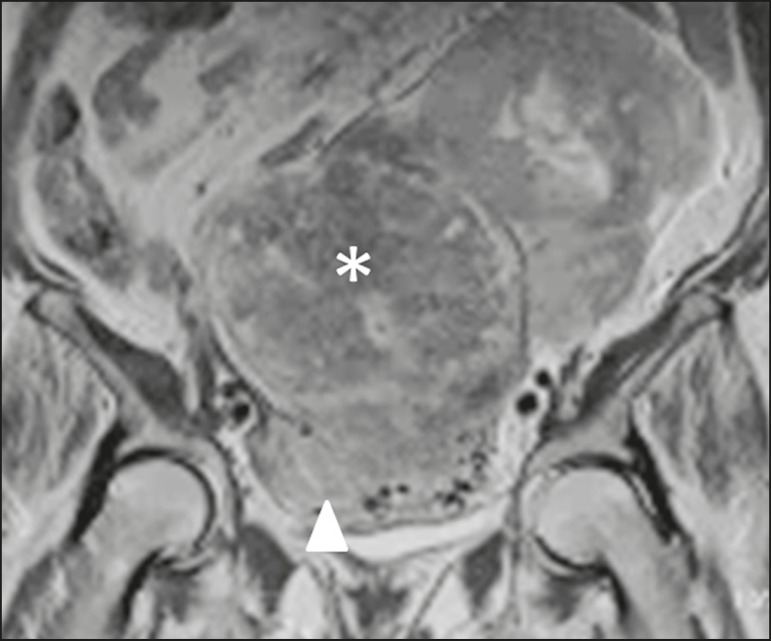
Leiomyosarcoma of the broad ligament in a 68-year-old woman. Coronal T2-weighted imaging showing a huge heterogeneous intermediate-signal solid mass in the pelvis (asterisk) with the uterus partly observed (triangle).

Primary leiomyosarcoma of the broad ligament (Figure 6) is extremely rare, with only a few cases reported in the medical literature. They are identical to the leiomyosarcomas observed in the uterus, presenting typically as large solitary masses with areas of hemorrhage and necrosis. Moreover, the criteria used for malignancy are similar with those used for leiomyosarcoma, with the following imaging findings: an irregular contour, high signal intensity on T2-weighted imaging, hyperintense areas on T1-weighted imaging, lack of calcifications, and restriction in diffusion WI, suggesting the nonspecific diagnosis^(4)^. It is suggested that the histopathological/molecular criteria for malignancy in a broad ligament leiomyosarcoma must be that of soft tissue, not the uterus^(2,6)^.

### Epithelial tumors of Müllerian type

**Figure 7:**
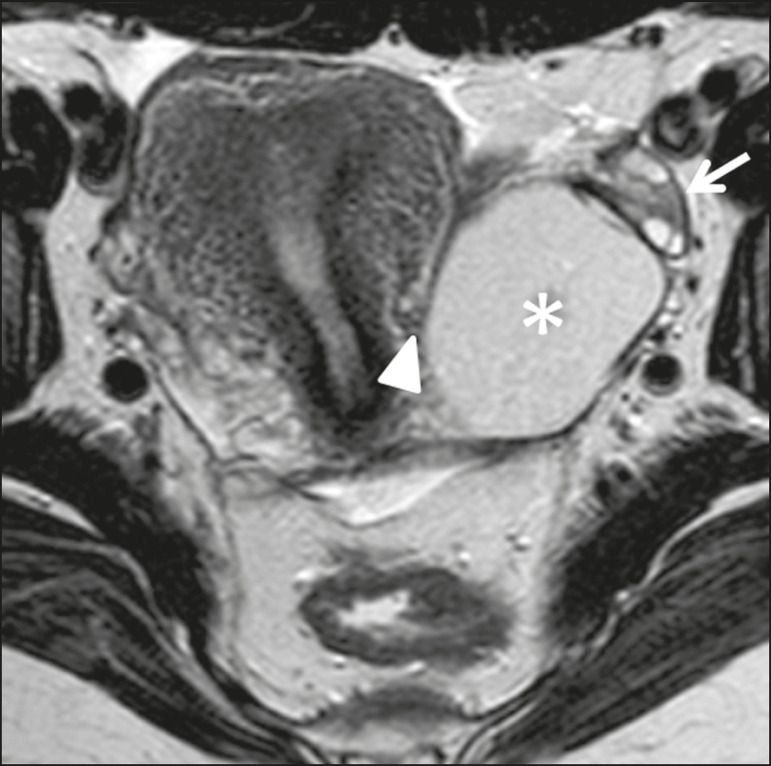
Serous cystadenoma of the broad ligament in a 19-year-old woman. Axial T2-weighted imaging showing a unilocular cystic tumor (asterisk) clearly demarked from the left ovary (arrow) and the uterus (triangle).

**Figure 8:**
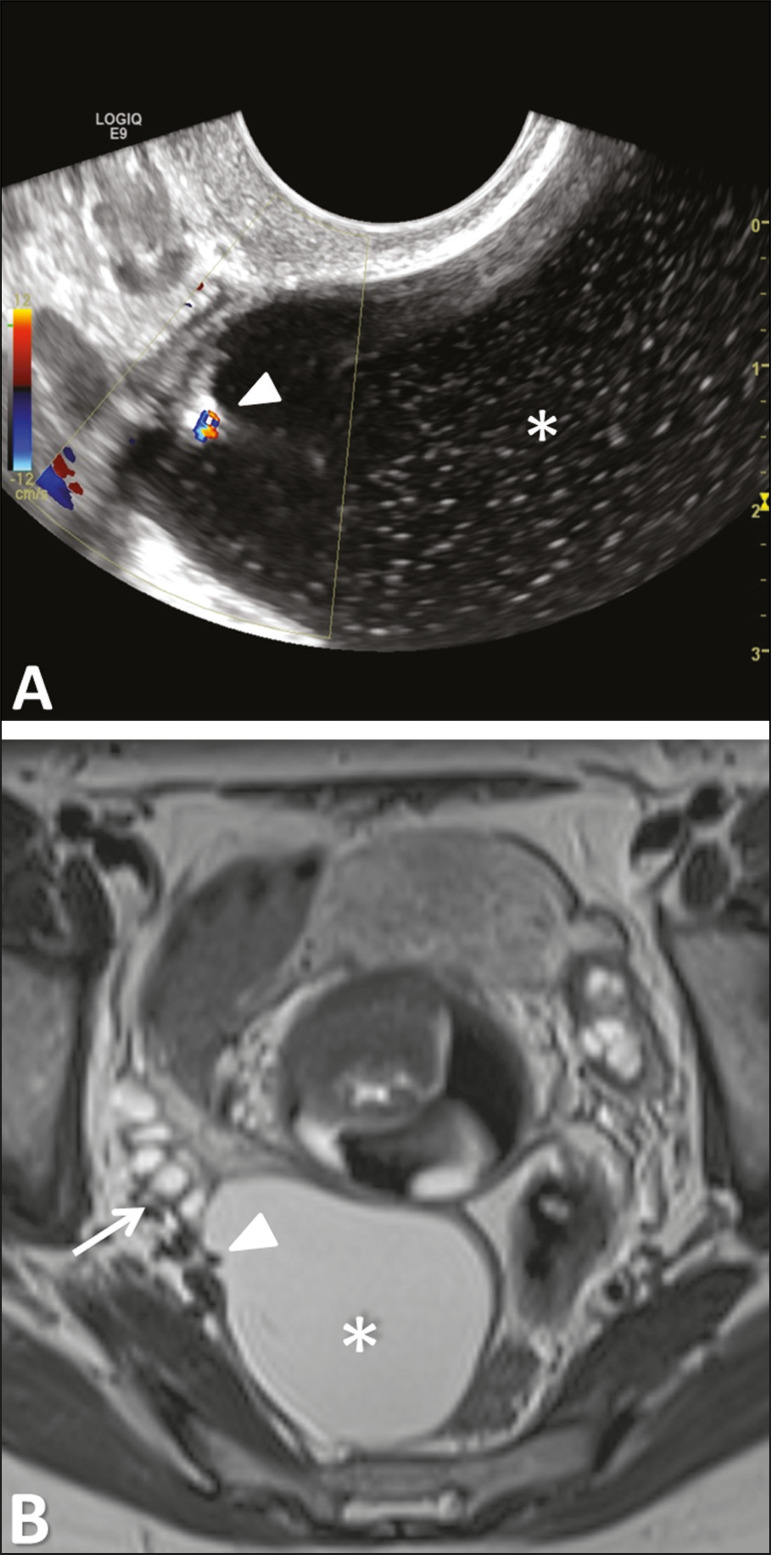
Serous borderline tumor of the left mesosalpinx in a 35-year-old woman. **A:** Transvaginal ultrasound showing a cystic tumor (asterisk) with a papillary projection (triangle) found to have vascularization with color Doppler. **B:** Axial T2-weighted imaging showing the cystic tumor with a single papillary projection, demarked from the right ovary (arrow).

These tumors are the most common type of epithelial neoplasm of the broad ligament and have the same multipotentiality to that of Müllerian epithelium elsewhere, ranging from benign, to borderline, to malignant. Paratubal/paraovarian broad ligament cysts represent approximately 10% of adnexal masses^(2)^ and tend to arise at a younger age than ovarian cysts. Nevertheless, paratubal/paraovarian broad ligament cysts and ovarian cysts have similar signs and symptoms. The most common one is *serous cystadenoma* (Figure 7), and most of these cystadenomas are unilateral, unilocular cysts up to several centimeters in diameter^(6)^. *Serous borderline tumor* (Figure 8), which is a tumor of low malignant potential with a histologic appearance intermediate between benign and malignant, may also arise as a primary tumor from the broad ligament, although with lesser incidence (2%) than the ovarian tumor (25%)^(7)^. Theories for the derivation of a serous borderline tumor in this location included invaginated fallopian tube epithelium, Müllerian remnant, or mesothelial origin^(2)^. Most patients diagnosed with serous borderline tumor were young, with only a few patients diagnosed with this tumor during the postmenopausal period^(7,8)^.

### Wolffian tumor/adnexal tumor of probable Wolffian origin

**Figure 9:**
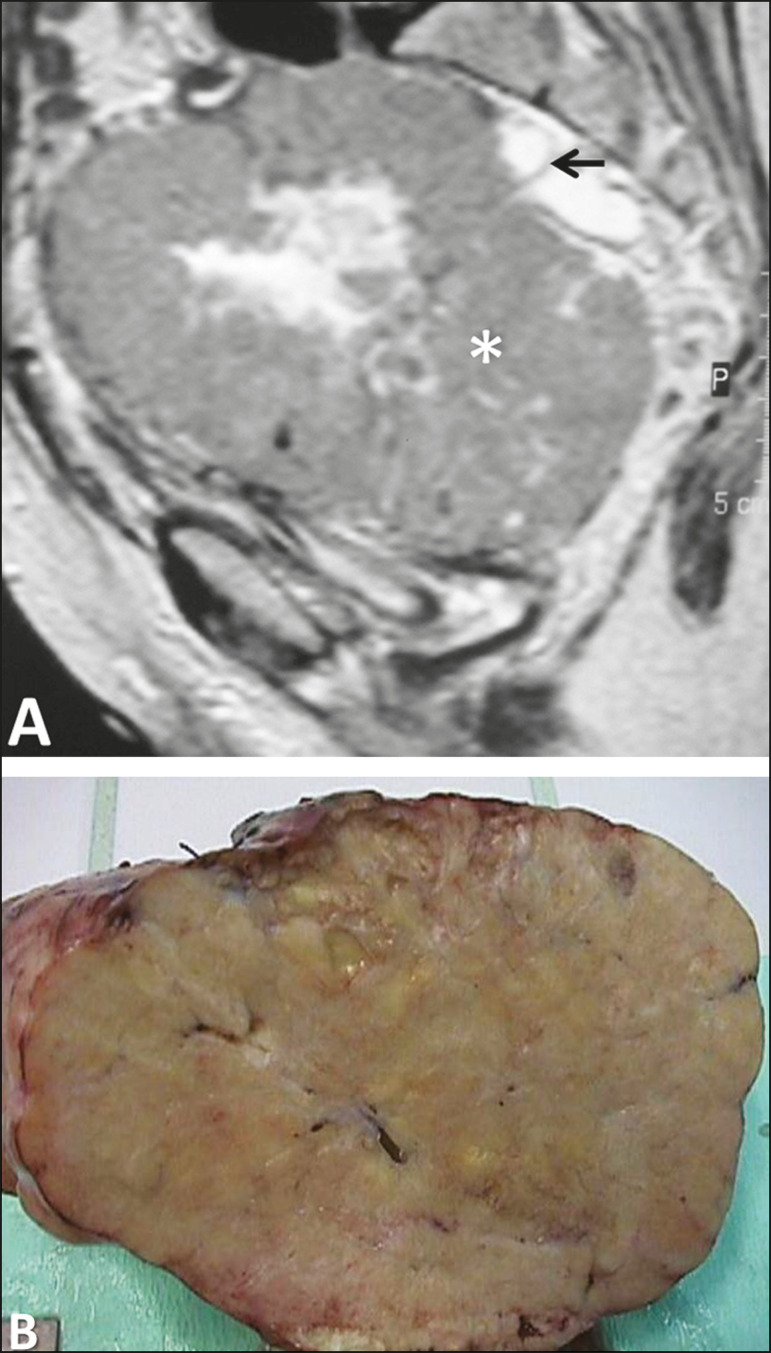
Adnexal tumor of probable Wolffian origin in a 69-year-old woman. **A:** Sagittal T2-weighted imaging showing a huge heterogeneous pelvic tumor, slightly hyperintense, with cystic degeneration (asterisk). The uterus is deviated anteriorly and to the left, and the ovaries are deviated backward (arrow). **B:** Gross specimen of the tumor.

Wolffian tumor/adnexal tumor of probable Wolffian origin (Figure 9) is a rare epithelial tumor of mesonephric origin reported to arise within the leaves of the broad ligament or suspended from it, from the remnants of the Wolffian duct, such as epoophoron, paroophoron, and Gartner’s duct. The median age of diagnosis for this tumor is 50 years, and over one-half of the cases are unilateral and incidental findings. Most cases are benign tumors, however, since some of them have aggressive courses, they are considered to have low malignant potential^(6,9)^.

### Papillary cystadenoma

**Figure 10:**
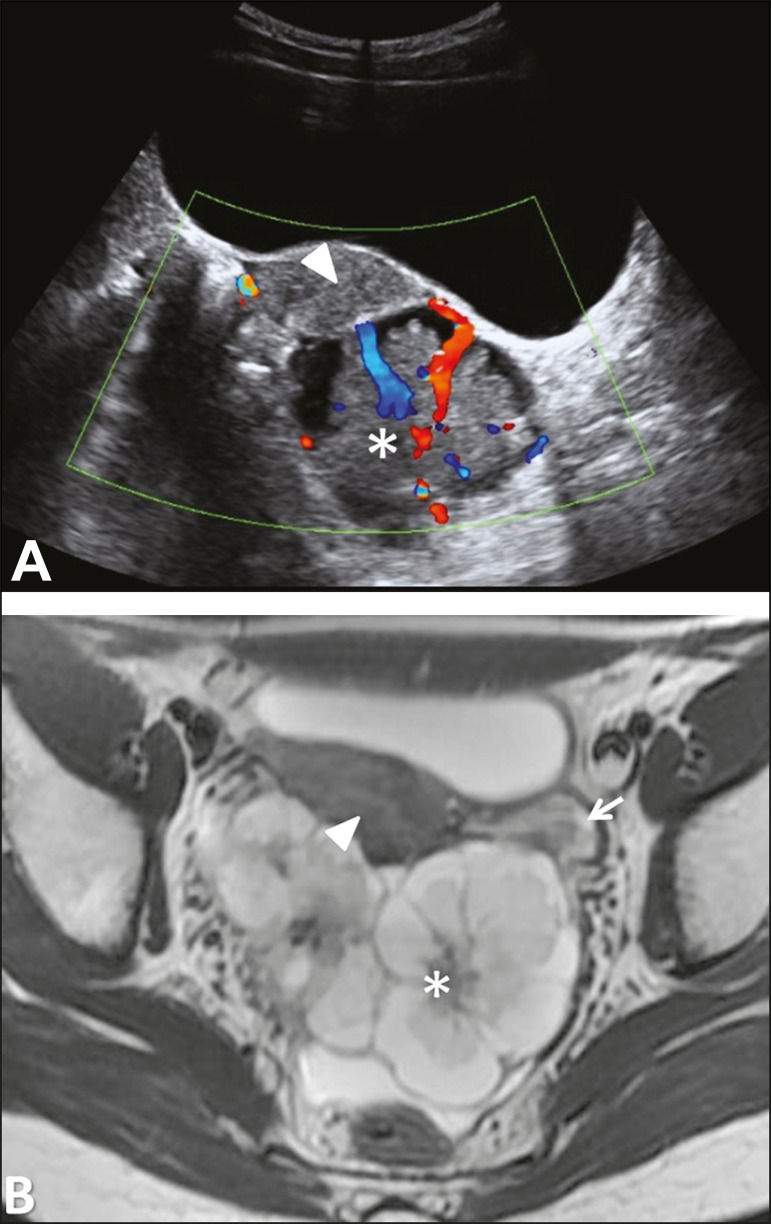
Papillary cystadenoma of the broad ligament in a 16-year-old patient with von Hippel–Lindau disease. **A:** Transvaginal ultrasound imaging showing a solid hypoechoic tumor with a cleavage plane with the uterus and color Doppler signal. **B:** Axial T2-weighted imaging demonstrating it as an intraperitoneal, retrouterine tumor, with mixed characteristics.

Papillary cystadenoma (Figure 10) is a rare, benign, cystic tumor considered to be of mesonephric origin and characteristically observed in patients with von Hippel-Lindau disease. These rare tumors have also been reported to occur in the epididymis and peritoneum, and in women, these tumors are found in the portion of the broad ligament near the fallopian tube (the mesosalpinx). The few reported cases of papillary cystadenoma are observed in younger women, and most of these cystadenomas have unilateral presentation^(6,8)^.

### Fibroma

**Figure 11:**
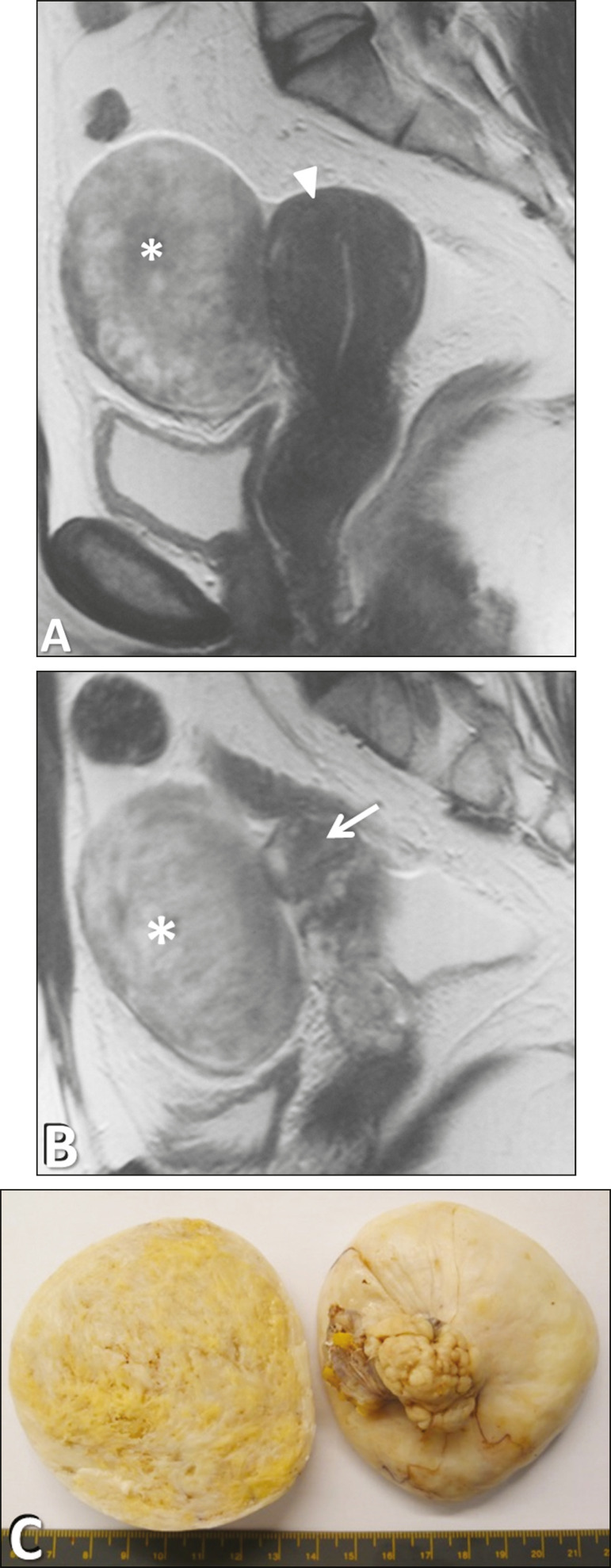
Cellular fibroma of the broad ligament in a 47-year-old woman. **A:** Sagittal T2-weighted imaging showing a mildly hyperintense solid tumor (asterisk) clearly demarked from the uterus (triangle). **B:** Sagittal T2-weighted imaging showing clear demarking of the tumor from the right ovary (arrow). **C:** Gross specimen of the tumor.

Fibroma (Figure 11), a benign pure stromal tumor, is common in the ovary, accounting for approximately 4% of all ovarian neoplasms, and is rarely observed in the broad ligament. It may be hardly differentiated from an ovarian tumor or a subserosal leiomyoma.

### Endometriosis

**Figure 12:**
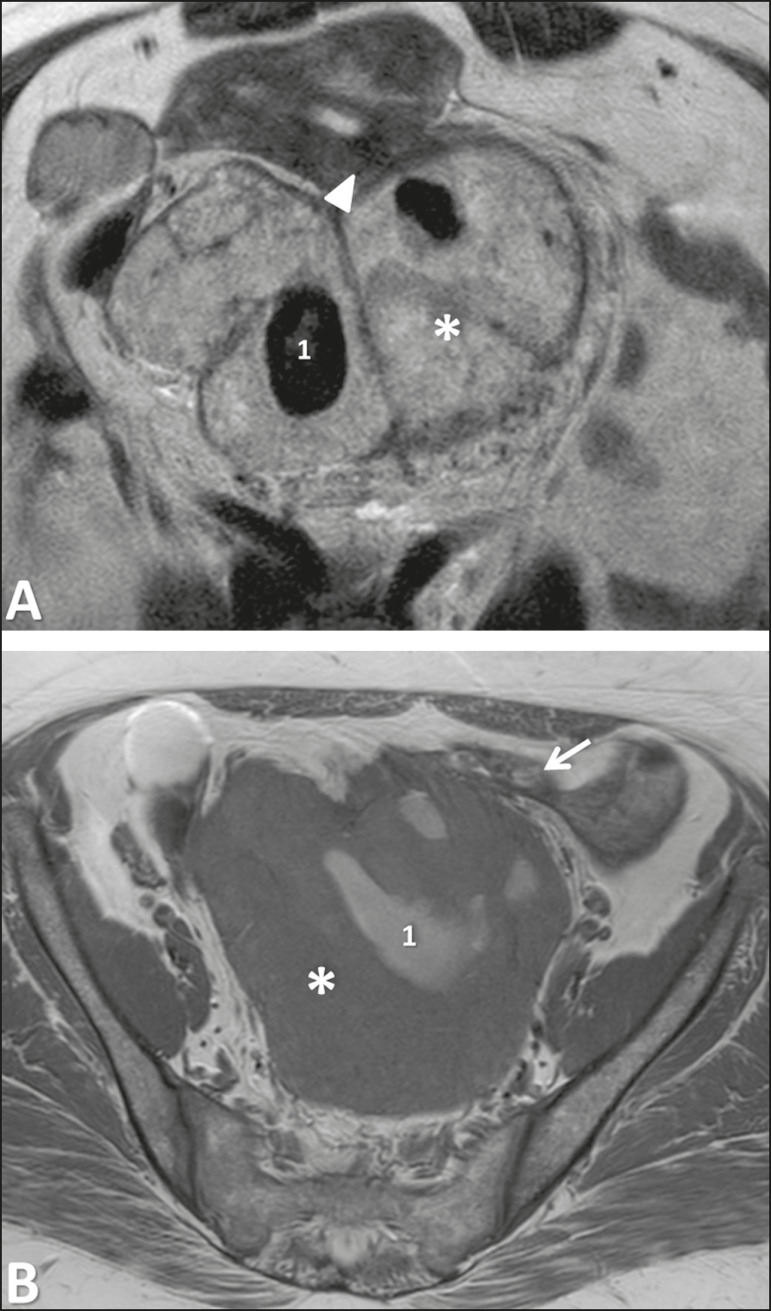
Endometriosis of the broad ligament in a 49-year-old woman. Axial T2-weighted imaging (**A**) and axial T1-weighted imaging (**B**) showing a heterogeneous tumor (asterisk), with internal areas with high T1 signal compatible with hemorrhagic areas (1), demarked from the left ovary (arrow) and the uterus (triangle).

Not every mass is a tumor, however, in the form of a tumor-like lesion, the presence of ectopically localized endometrial tissue-known as endometriosis-in the broad ligament is common (Figure 12).

### Müllerianosis

**Figure 13:**
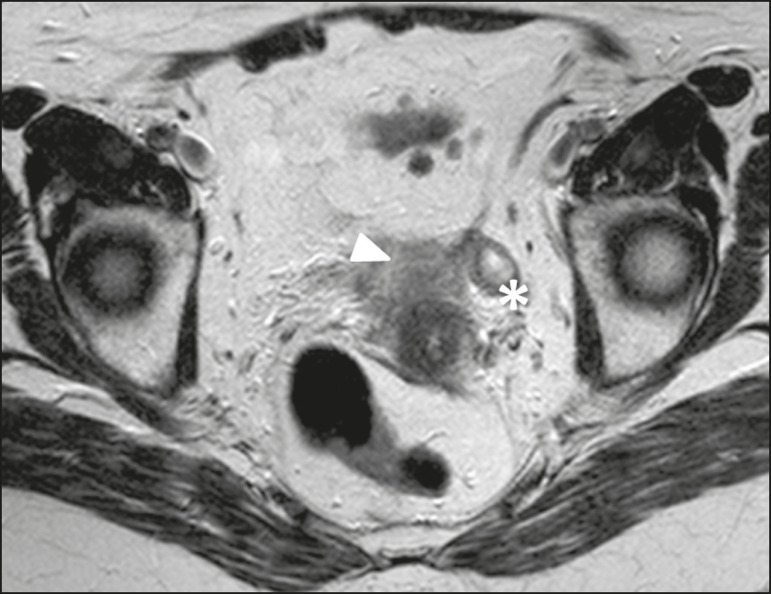
Müllerianosis of the left broad ligament in a 74-year-old woman. Axial T2-weighted imaging showing a tumor with similar characteristics with the uterus (asterisk) suspended from the broad ligament and clearly demarking the uterus (triangle).

Müllerianosis (Figure 13), a benign tumor-like lesion different from endometriosis considering that at least two types of Müllerian-derived tissue are ectopically located through a developmental anomaly, which is considered a rare type of choristoma, can also occur in the broad ligament. To establish the diagnosis of müllerianosis, the patient should have no evidence of pelvic endometriosis, no history of surgery to the reproductive organs, and no direct communication of the lesion with the endocervix, endometrium, or endosalpinx^(10)^. The lesion is frequently observed in association with borderline ovarian neoplasms. Hence, it is important not to interpret the finding as metastatic^(3)^.

### Secondary malignancies

Secondary malignancies of the broad ligament include metastatic disease from endometrial, cervical, and ovarian carcinoma and other tumors not arising in the female reproductive organs^(6)^.

## CONCLUSION

Primary tumors of the broad ligament are rare, although its involvement by malignant tumors arising elsewhere in the abdomen and pelvis is common. These can be histologically similar to tumors observed in the uterus and the ovaries or can be different due to the presence of embryonic remains particular to this location. Thus, it is important for a radiologist to acknowledge the potential range of lesions from this region and recognize some of the imaging findings that may suggest such location, considering that one appropriate diagnosis may influence its management.
